# Sweet Electronics: Honey‐Gated Complementary Organic Transistors and Circuits Operating in Air

**DOI:** 10.1002/adma.202103183

**Published:** 2021-08-21

**Authors:** Alina S. Sharova, Mario Caironi

**Affiliations:** ^1^ Center for Nano Science and Technology @PoliMi Istituto Italiano di Tecnologia Via G. Pascoli, 70/3 Milano 20133 Italy; ^2^ Department of Physics Politecnico di Milano Piazza Leonardo da Vinci, 32 Milano 20133 Italy

**Keywords:** edible electronics, electrolyte‐gated transistors, honey, organic electronics, printed electronics

## Abstract

Sustainable harnessing of natural resources is key moving toward a new‐generation electronics, which features a unique combination of electronic functionality, low cost, and absence of environmental and health hazards. Within this framework, edible electronics, of which transistors and circuits are a fundamental component, is an emerging field, exploiting edible materials that can be safely ingested, and subsequently digested after performing their function. Dielectrics are a critical functional element of transistors, often constituting their major volume. Yet, to date, there are only scarce examples of electrolytic food‐based materials able to provide low‐voltage operation of transistors at ambient conditions. In this context, a cost‐effective and edible substance, honey, is proposed to be used as an electrolytic gate viscous dielectric in electrolyte‐gated organic field‐effect transistors (OFETs). Both n‐ and p‐type honey‐gated OFETs (HGOFETs) are demonstrated, with distinctive features such as low voltage (<1 V) operation, long‐term shelf life and operation stability in air, and compatibility with large‐area fabrication processes, such as inkjet printing on edible tattoo‐paper. Such complementary devices enable robust honey‐based integrated logic circuits, here exemplified by inverting logic gates and ring oscillators. A marked device responsivity to humidity provides promising opportunities for sensing applications, specifically, for moisture control of dried or dehydrated food.

## Graphical abstract

The development of edible electronics devices requires unconventional solutions, in particular, the use of functional food‐based materials in the design. An inherently safe and nutritive compound, honey, is hereby proposed as an effective gating medium for the realization of air‐stable complementary organic transistors and circuits operating at low voltage (<1 V) and featuring other distinctive electronic functionalities.

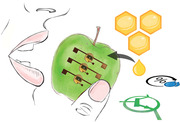

## 1 Introduction

Steering the technological development in a sustainable direction has become mandatory. The acknowledgment of thºe global problem of electronic and plastic waste and its consequences, posing severe risks to the environment and human health, asks for drastic countermeasures. For this reason, the demand for readily available natural resources for the design and fabrication of “benign” electronic solutions is growing. As a result, recently, an increased emphasis has been placed on the investigation of alternative organic, green, and biodegradable electronics.^[^ ^1^, ^2^, ^3^ ^]^

When the electronic technology faces the healthcare and food sector, safety of the devices becomes mandatory. The latter is particularly critical when the electronic systems are intended to explicitly interact with the inside of the human body, be ingested either along with the consumed food or pharmaceutical products. In this framework, ingestible electronics has so far achieved remarkable advances paving the way for a new era diagnostics and therapy.^[^ ^4^, ^5^, ^6^, ^7^, ^8^ ^]^ However, ingestible systems available to date,^[^ ^9^ ^]^ apart from their bulk design and need of post‐performance recollection, have critical drawbacks manifesting primarily in the use of toxic and non disposable materials, posing hazards not only to the consumer health but also to the environment.

To this end, the recently conceptualized “edible electronics”^[^ ^10^, ^11^, ^12^ ^]^ envisions electronic systems fulfilling key electronic functionalities, being sustainable, nontoxic, safe for ingestion, and cost‐effective at the same time. The unique feature of this emerging field lies in exploiting edible materials of different nature (e.g., food, drugs, edible metals, edible pigments, dyes, and polymers) as electronics constituents, according to their electronic properties, to provide all the necessary building blocks: conductors, insulators, semiconductors. Due to the absolute safe composition, edible devices are intended to undergo degradation within the body after accomplishing their task, what implies elimination of any potential adverse effects.

Being at an emerging stage, the field is scarce in examples. Yet the feasibility of this new paradigm relies on several inspiring and rather exotic prototypes of edible, and in particular food‐based electronic components, such as cheese supercapacitors, ^[^ ^13^ ^]^ broccoli microphones, ^[^ ^14^ ^]^ charcoal‐based biofuel cells, ^[^ ^15^ ^]^ silk sensors, ^[^ ^16^ ^]^ transistors based on edible pigments, ^[^ ^12^, ^17^ ^]^ among others.

In order to fulfill the fundamental electronic duties of tracking, monitoring, sensing, and data transmission, edible electronics systems will require active circuits. In this context, transistors represent the backbone components of future edible systems, for which low‐voltage/low‐power operation is mandatory.

Edible transistors are exemplified so far by only few demonstrations realized primarily through vacuum‐based thermal evaporation^[^ ^12^ ^]^ or printing techniques.^[^ ^18^ ^]^ By employing edible dielectrics (e.g., guanine, adenine, albumin, ethilcellulose, among others^[^ ^12^, ^18^, ^19^, ^20^ ^]^), field‐effect transistors (FETs), either entirely or partially edible, were demonstrated, with operational voltages in the range from 5 to 60 V.

To lower the operation voltage and meet requirements of edible electronics, electrolyte‐gated organic FETs (EGOFETs) appear as a powerful option. In fact, because of very large typical areal capacitance (µF cm^−2^),^[^ ^21^ ^]^ operational voltages <1 V can be adopted. Moreover, EGOFETs provide coupling of both ionic and electronic domains in a single device, what opens various opportunities for sensing applications.^[^ ^22^, ^23^, ^24^, ^25^ ^]^ Among widely investigated electrolytic compounds (e.g., ionic liquids, ion‐gels, aqueous and solid‐state electrolytes),^[^ ^26^, ^27^, ^28^, ^29^ ^]^ pure/distilled water is a common gating media.^[^ ^30^, ^31^, ^32^, ^33^ ^]^ While aqueous gating is being exploited for biosensing assays,^[^ ^34^, ^35^, ^36^ ^]^ a liquid gating phase clearly poses issues in circuits integration.

A strategy to overcome this limitation is represented by the exploitation of electrolytic properties of food stuff to implement edible gating media in EGOFETs. Herein, we investigate electrical performance of a low cost, widely accessible and edible by definition substance, honey, as an electrolytic gate dielectric within the EGOFET architecture. Honey is recognized as a supersaturated viscous electrolytic solution that contains mainly sugars (>80 wt%; fructose, glucose, sucrose), water (<17 wt%), and minor components (enzymes, amino and other acids, lipids, proteins, vitamins, minerals, and others) in varying quantities depending on the honey origin and environmental conditions.^[^ ^37^ ^]^ Besides showing ionic conductivity related to its hydration state,^[^ ^38^ ^]^ honey is characterized by intrinsic properties of long shelf life, high viscosity, hygroscopicity, antioxidant capacity, and antibacterial activity,^[^ ^39^, ^40^ ^]^ what makes this nutritive sweet compound an appealing candidate for applications in convergence of healthcare, biology, and electronics.

The approach of using honey as electrolyte gate dielectric has been so far proposed only to gate graphene for a rapid determination of its quality.^[^ ^41^ ^]^ No honey‐gated devices or integrated circuits based on organic and edible materials, have been, however, reported until now.

In this work, we demonstrate air‐stable, low‐voltage, p‐type and n‐type honey‐gated OFETs (HGOFETs), and their combination in complementary inverting logic gates and ring oscillators. We also show the compatibility of our HGOFETs with fabrication on an edible flexible tattoo‐paper substrate, which could be conformally adhered to different surfaces, in particular, food items. The introduction of honey as a nontoxic, commercially available, cost effective, and easily processable electrolyte gate dielectric is potentially advantageous for various edible electronics applications ranging from biomedicine to food industry.

## 2 Results

**Figure 1:**
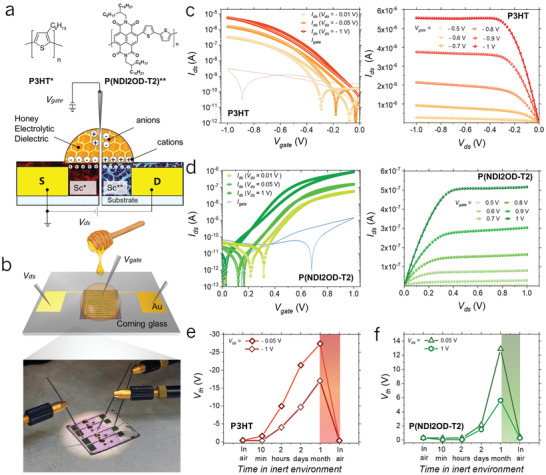
HGOFETs structure, fabrication, and electrical characterization. a) A schematic cross‐section of the p‐type (left) and n‐type (right) HGOFETs with the representation of a simplistic charge carriers distribution and molecular structures of the exploited semiconductors: P3HT and P(NDI2OD‐T2). b) Illustration (top) and a digital image (bottom) of the HGOFET with gold source and drain electrodes on glass, spin‐coated semiconductor, and drop‐cast honey (pink color corresponds to the spin‐coated P3HT thin film). c) Transfer (left) and output (right) characteristic curves for p‐type HGOFETs in linear (*V*_ds_ = −0.01 V; −0.05 V) and saturation (*V*_ds_ = −1 V) regimes; *V*_gate_ sweep rate was 5 mV s^−1^. d) Transfer (left) and output (right) characteristic curves for n‐type HGOFETs in linear (*V*_ds_ = 0.01 V; 0.05 V) and saturation (*V*_ds_ =1 V) regimes; *V*_gate_ sweep rate was 2 mV s^−1^; OFETs geometrical parameters: *L* =10 µm; *W* = 20 000 µm. e,f) The reversible electrical response of HGOFETs to different humidity conditions (RH in air ≈50%; in inert nitrogen environment ≈0%). The humidity‐dependent behavior of *V*_th_ for the p‐type (e) and n‐type (f) HGOFET operating in both linear (*V*_ds_ = ± 0.05 V) and saturation (*V*_ds_ = ±1 V) regimes. The rectangular regions correspond to the hydration of the dehydrated devices right after exposing them to air.

In order to verify the intrinsic electronic performance of honey as a gating media for polymer semiconductors, a first set of HGOFETs was realized on rigid glass substrates, serving as a reference. A schematic cross‐section of top‐gate bottom‐contact HGOFETs configuration is shown in **Figure** **1**a. Gold source and drain electrodes were patterned by photolithography, with the channel length *L* and the channel width *W* of 10 µm and 20 000 µm, respectively.

Given the substantial lack of proved edible, high‐performance solution processible organic semiconductors, two well‐known model polymers were used in our tests to demonstrate the compatibility of HGOFETs with both hole and electron transporting materials. One of the most extensively studied good hole transporting polymer semiconductors, poly(3‐hexylthiophene) (P3HT), the biocompatibility of which has been widely recognized,^[^ ^42^, ^43^, ^44^ ^]^ was employed for p‐type HGOFETs. Poly{[*N*,*N*′‐bis(2‐octyldodecyl)‐naphthalene‐1,4,5,8‐bis(dicarboximide)‐2,6‐diyl]‐*alt*‐5,5′‐(2,2′‐bisthiophene)} (P(NDI2OD‐T2)), an intensively investigated electron‐transporting copolymer, was, on the other hand, used in n‐type devices. In terms of biocompatibility, the latter has been so far subjected to cytotoxicity tests only, with no adverse effects detected for proliferation of line cells such as HUTU‐80 and CACO‐2.^[^ ^18^ ^]^

The aforementioned P3HT and P(NDI2OD‐T2) polymers were spin‐coated onto the substrates provided with the interdigitated contacts, and a drop of commercially available honey (see the Experimental Section for details) was than cast on the semiconductor surface at ambient conditions (Figure 1a,b). A tungsten tip was subsequently dipped in honey to serve as a gate electrode.

When an external negative voltage is applied to the gate tip for p‐type operation, cations accumulate at the gate/honey interface, while anions are repelled toward the honey/semiconductor interface, producing the accumulation of holes in the semiconductor. The reverse charge distribution occurs in n‐type gating, when the positive potential is applied to the gate.

The current–voltage (*I*–*V*) transfer and output characteristics of the p‐type and n‐type devices are given in Figure 1c,d, correspondingly. The curves presented demonstrate a typical FET operation in both linear (drain–source voltage *V*_ds_ of ±10 mV and ±50 mV) and saturation regimes (*V*_ds_ = ±1 V) at absolute gate voltages *V*_gate_ below 1 V. The threshold voltage *V*_th_ was found to be ≈0.25 V for P(NDI2OD‐T2)‐based HGOFETs and ≈−0.35 V for P3HT‐based HGOFETs at *V*_ds_ = ±1 V. The transfer voltage sweeps exhibit a modest hysteresis and a more evident *V*_ds_ dependent threshold voltage shift, which might be related to field‐dependent charge injection.^[^ ^45^, ^46^ ^]^ A distinctive feature of the demonstrated HGOFETs is their reproducibility (Figure S1, Supporting Information) and stable operation in air, at room temperature (relative humidity, RH, of about 50%). Devices revealed a shelf‐life stability up to 1 month control period, while being stored in air (Figure S2, Supporting Information), with no evidence of electronic performances degradation (RH varied from 48.8% to 50.4%). The shelf life of HGOFETs was limited by the time of naturally occurring honey crystallization that exceeded in our case the control period of 1 month. Operational stability of the devices has been as well preliminarily validated through a continuous cycling voltage test (30 min total duration) performed in air by switching the devices on and off (Figure S3, Supporting Information).

Interestingly, the OFETs based on the chosen polymer semiconductors typically show a poor air stability. In particular, P3HT is known to be subjected to photodoping upon exposure of the material to light in air,^[^ ^35^, ^47^, ^48^, ^49^, ^50^ ^]^ which can drastically limit charge modulation and the on/off current ratio of the device. P(NDI2OD‐T2) charged with an excess electron undergoes, on the other hand, oxygen and water‐induced reversible and irreversible degradation processes.^[^ ^51^, ^52^, ^53^ ^]^ The drastically improved ambient stability conferred by honey, cast onto the semiconductors surface, indicates that it additionally acts as an encapsulant forming a kinetic barrier against diffusion of water and oxygen into the active channel region. The origin of this effect requires more detailed investigation, but is likely related to the low water activity of honey, of about 0.6, and its high osmotic pressure.^[^ ^39^, ^40^ ^]^ Fresh fruits and vegetables, e.g., have water activity of around 0.99, whereas the water activity of pure water is 1.^[^ ^54^ ^]^ We anticipate that the specific viscosity of the selected honey may modulate gating, as well as barrier properties.^[^ ^55^ ^]^

As a characteristic figure of merit of electrolyte‐gated transistors, the mobility–capacitance product μ_sat_·*C* has been extracted for HGOFETs from the transfer characteristic curves in the saturation regime according to:

(1) $$\mu_{\text{sat}}\cdot C=\frac{2L}{W}{\left(\frac{\partial \sqrt{I_{\text{ds}}}}{\partial V_{\text{gate}}}\right)}^{2}$$

where μ_s_ is the mobility in the saturation regime, *C* is the gate capacitance per unit area, *I*_ds_ is the drain current, and *V*_gate_ is the gate voltage. The determined μ_sat_·*C* values are ≈23 nF V^−1^ s^−1^ for p‐type and ≈3.5 nF V^−1^ s^−1^ for n‐type HGOFETs. Both values are one order of magnitude higher than those previously obtained in water‐gated organic FETs (WGOFETs), with identical architecture, based on the same polymers and characterized within the same range of voltages (**Table** **1**). In order to make a demonstrative comparison of the two configurations, the areal capacitance values *C* of metal–insulator–semiconductor (MIS) structures, consisting of Au/honey/semiconductor/Au, were evaluated at 0.1 Hz from electrical impedance spectroscopy (EIS) measurements (Figure S4, Supporting Information, average impedance with standard deviation among three samples). *C* was found to be 2.3 µF cm^−2^ for the P3HT‐based MIS with the polymer thickness of 35 nm, and 1 µF cm^−2^ for the P(NDI2OD‐T2) one with the polymer thickness of 32 nm. The capacitance values obtained are compatible with an interface field‐effect, rather than a volumetric one, suggesting that ions from the honey do not penetrate into the bulk of the semiconductor. The formation of an electrical double layer is in agreement with previously reported studies on WGOFETs involving the same hole transporting material^[^ ^56^ ^]^ and additional experiments realized for honey‐based devices with different semiconductor thicknesses (Figures S5 and S6, Supporting Information). Additionally, we observed that the electrical properties of HGOFETs and HGOFETs‐based circuits were not affected by the quantity/thickness of the honey drop when hydrated. The depth of the gate electrode immersed in honey neither influenced the devices electrical performance. The average volume of the honey drop was 20 µL, however, significantly varied from device to device without affecting their input or output electrical characteristics (Figure S1, Supporting Information). This is consistent with the fact that the electric double layer develops at an interface, and its formation should not depend on the electrolyte thickness. We, therefore, expect no capacitance dependence on the quantity of the honey drop.

**Table 1:** The comparison of the electrical characteristics (areal capacitance *C*, mobility‐capacitance product μ_s_·*C*) between honey‐gated and previously reported water‐gated transistors. The capacitance values are extracted from EIS measurements at 0.1 Hz

	*C* [µF cm^−2^]	μ_s_·*C* [nF V^−1^ s^−1^ ]
P3HT_WGOFET_^[^ ^58^ ^]^	2	6
P3HT_HGOFET_	2.3	23
P(NDI2OD‐T2)_WGOFET_^[^ ^59^ ^]^	0.08	0.3
P(NDI2OD‐T2)_HGOFET_	1	3.5

We attribute the electrolyte gating behavior of honey to its marked hygroscopic nature, i.e., its ability to absorb/reabsorb moisture from the surrounding environment due to the low water activity. In this scenario, at ambient conditions, the movement of ionic honey species within the honey matrix is enabled by presence of water. The diffusion of ions within honey under the influence of the applied electrical field is, hence, directly dependent on the honey moisture content. The likelihood of hydrolysis of water present in honey, which might drastically impact the devices performance, is eliminated since the applied voltage range does not exceed the minimum voltage stimulating water decomposition equal to 1.229 V at 25 °C.^[^ ^57^ ^]^

Given the above picture, hydration of honey is critical to its gating properties. As a further confirmation, we tested the effect of honey dehydration by transferring HGOFETs devices to an inert nitrogen environment, with RH ≈ 0%. As a consequence of storing the devices in inert atmosphere, a first significant threshold voltage *V*_th_ increase for both the p and n‐type HGOFETs was registered within hours (Figure 1e,f). Over a period of 1 month, *V*_th_ reached equilibrium, with an overall increase of two orders of magnitude with respect to its value for hydrated honey, reaching −28 and 13 V in saturation for p‐ and n‐type HGOFETs, respectively. In the absence of absorbed water, ionic conductivity is compromised and the capacitance is drastically reduced (*C* = 0.05 nF cm^−2^), to a level expected in non‐electrolytic dielectrics, with a corresponding need of very high voltages to accumulate an electronic channel in the semiconductors (Figure S7, Supporting Information). Once honey is rehydrated, by transferring the devices back to ambient atmosphere (RH ≈ 50%), HGOFETs recover the original low voltage operation as an effect of electrolyte‐gating within few minutes (Figures S8 and S9, Supporting Information).

**Figure 2:**
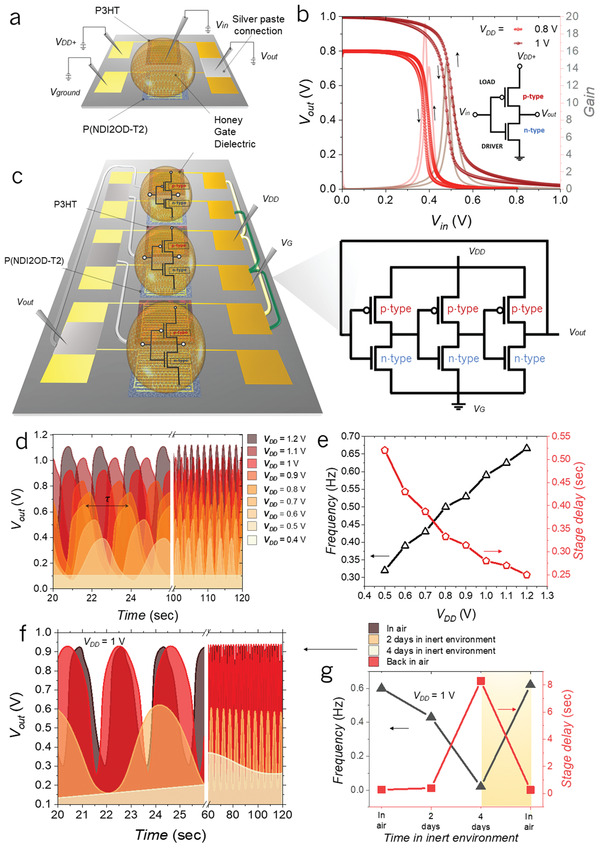
HGOFETs‐based logic circuits and their electrical characterization. a) Schematic representation of the inverting logic gate device realized with P3HT and P(NDI2OD‐T2). b) VTCs, and corresponding derivative curves to extract gain, of a complementary HGOFETs‐based inverter as a function of input voltages; the arrows indicate the sweep (rate of 25 mV s^−1^) direction; the inverter configuration is reported as an inset. c) Schematic representation (left) and the circuit configuration (right) of the three‐stage HGOFETs‐based ring oscillator. d) Output waveforms of the HGOFETs‐based oscillator operating in air at different supply voltages *V*_DD_. e) Output oscillation frequency (black line) and propagation signal delay per stage (red line) of the HGOFETs‐based oscillator operating in air as a function of *V*_DD_. f) Output waveforms of the HGOFETs‐based oscillator at different humidity conditions (in air; in inert environment) at *V*_DD_ = 1 V. g) Output oscillation frequency (black line) and stage delay (red line) of the HGOFETs‐based oscillator as a function of the humidity condition. The rectangular region schematically corresponds to the hydration of the dehydrated device right after exposing it to air.

The demonstrated operability of HGOFETs and the possibility to realize both n‐type and p‐type devices allow the realization of complementary logic circuits, the most robust logic circuits topology, gated through honey. We first implemented a complementary logic inverter based on the aforementioned HGOFETs. **Figure** **2**a reports a visual representation of a honey‐based complementary inverter, the geometrical parameters of which were chosen to balance the currents of the n‐ and p‐type devices (p‐type: *L* = 20 µm, *W* = 20 000 µm; n‐type: *L* = 10 µm, *W* = 20 000 µm). As a result, a balanced device operation with symmetrical voltage‐transfer characteristics (VTC) and negligible hysteresis was obtained (Figure 2b). The inversion threshold (*V*_inv_) is correctly placed at half of the supply voltage (*V*_DD_/2), being ≈0.5 V at *V*_DD_ = 1 V and ≈0.4 V at *V*_DD_ = 0.8 V. The inverter shows a high gain of ≈−19 at the logic transition at *V*_DD_ = 0.8 V.

The correct operation of the honey‐gated inverters allowed us to combine them in a ring oscillator (Figure 2c), to demonstrate the use of HGOFETs in complementary circuits. The output signal of a three‐stage honey‐based ring oscillator at various *V*_DD_ is reported in Figure 2d,e. The increase of the device oscillation frequency (*f*_osc_) from 0.32 to 0.67 Hz, along with the decrease of the stage delay from 0.53 to 0.25 s, is observed with raising *V*_DD_ from 0.4 to 1.2 V. The ring oscillator itself is proved to be sensitive to the hydration of honey. In fact, its electrical parameters, such as the oscillation amplitude and *f*_osc_ drastically change from ambient (RH ≈ 50%) to inert atmosphere (RH ≈ 0%, Figure 2f). In particular, *f*_osc_ measured at a fixed *V*_DD_ = 1 V decreases from 0.6 Hz in ambient atmosphere to 0.02 Hz after 4 days of storage in inert atmosphere (Figure S10, Supporting Information). The reverse process, with *f*_osc_ rising again to 0.62 Hz, occurs within minutes after re‐exposing the circuit to air.

The control of relative humidity can be critical for many applications, in particular, for food and beverages or healthcare industries, where the quality of the products can strongly depend on the moisture content of the atmosphere they are exposed to. Therefore, targeting the concept of safe and edible electronic systems, HGOFETs and HGOFETs‐based circuits could potentially be integrated as reversible humidity indicators of humidity/dry conditions, more specifically, for moisture monitoring of dried or dehydrated food (e.g., food packaging).

On the other hand, the humidity‐dependent behavior of honey may pose a stability issue for circuits. Monitoring the devices in ambient conditions at varied humidity over a month period (Figure S2, Supporting Information), we observed that within an RH range of 45–60% no major variations in electrical properties of the devices occur, thus indicating that the gating properties of honey are preserved. This is in agreement with previous studies reporting no drastic variation of moisture content in honey in the same RH range.^[^ ^60^ ^]^ When such RH conditions cannot be ensured, to achieve stability on a broader range of humidity values, the moisture content of honey could be potentially preserved by, e.g., encapsulating it with beeswax, an authorized food additive (E901). Such encapsulation would replicate the honeycomb, which is naturally intended for honey moisture content preservation.

Finally, as a proof of concept, we aimed at demonstrating the possibility to transfer the air‐stable low‐voltage HGOFETs on edible items, in particularly food, by exploiting temporary tattoo‐paper substrates.^[^ ^18^ ^]^

To this end, the interdigitated source and drain electrodes of the HGOFETs (*L* = 100 µm, *W* = 20 000 µm) were patterned through thermal shadow‐mask evaporation of gold onto the ethylcellulose (EC) surface of the tattoo‐paper. The biocompatible P3HT active layer was inkjet‐printed, and the honey was then drop‐cast to cover the HGOFETs channel area.

**Figure 3:**
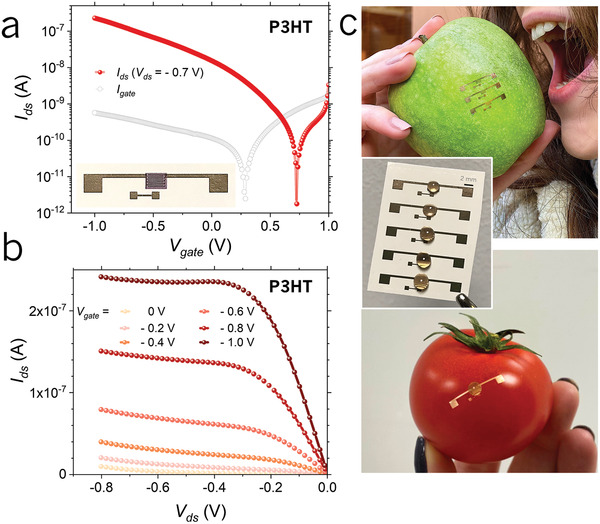
HGOFETs on flexible edible tattoo‐paper substrate: electrical characterization and devices conformability. a) Transfer in saturation (*V*_ds_ = −0.7 V) regime and b) output characteristic curves for p‐type HGOFET after the transfer onto the apple surface. c) Digital photograph of HGOFETs (in the middle), HGOFETs transferred onto different food items: an apple (top) and tomato (bottom).

The transfer of the devices was achieved by aqueous dissolution of the sacrificial starch/dextrin layer, and release of the sub‐micrometric film of EC onto the target item by sliding it off the paper backing. The electrical characterization in saturation regime (*V*_ds_ = −0.7 V) shows that the tattoo‐paper approach allows the correct low voltage (*V*_gate_ < 1 V) operation of the p‐type HGOFETs before (Figure S11, Supporting Information) and after (**Figure** **3**a,b) the demonstrative device transfer onto the apple skin. The threshold voltage of the transferred device was found to be ≈0.75 V at *V*_ds_ = −0.7 V. Our experiments overall demonstrate that by means of untreated commercial tattoo‐paper, HGOFETs can be easily deployed onto various surfaces, in particular, food items (Figure 3c).

The final configuration of the HGOFET employs to a greater extent edible nature‐derived and commodity materials in the design. The device was assembled on the substrate of a cellulose derivative, EC, a food additive (E462) approved by the US Food and Drug Administration (FDA) and widely used as a coating agent, emulsifier, binder or filler in the pharmaceutical, cosmetics, and food technologies. The electrically conductive components of the device were realized in gold, a biologically inert stable substance. With a purity higher than 23 karat, gold is accepted in food industry as garnish to various food items (E175). Honey dielectric is an inherently nutritious edible compound that, owing to its complex chemical composition and physical properties, finds wide range applications not just in the food sector but also in medicine. The compatibility of the adopted semiconducting polymers with ingestion, on the other hand, is the most critical requirement for the overall edibility of the final device, in spite of the extremely low quantity of active material exploited for the fabrication of a single HGOFET, in the range of picograms.^[^ ^18^ ^]^ Although P3HT has been recognized as a biocompatible material, and P(NDI2OD‐2T) has been previously subject to cytotoxicity studies on cell lines derived from human GI tissue, a more profound complex analysis of polymers edibility is still needed, which will eventually require clinical tests. To prepare the ground for future edibility studies, we are currently applying in vitro digestion protocols to simulate the physiological conditions of devices during in vivo digestion and first assess safety, with special emphasis on the semiconducting materials.

## 3 Conclusion

We have presented the use of honey, a naturally occurring and inexpensive food, as an electrolytic gate dielectric for organic transistors. Honey‐gated complementary polymer transistors and logic circuits, namely, inverting logic gates and ring oscillators, operating in ambient air at low voltages, <1 V, were demonstrated.

Devices exhibit promising electronic performances in terms of mobility‐capacitance product, negligible hysteresis, competing with or outperforming previously reported water‐gated systems, based on the same polymers. We related the excellent gating features to the hydration of honey, which thanks to a rather low water activity absorbs water from the environment. Such characteristic donates to HGOFETs a strong sensitivity to relative humidity levels, suggesting a possible use as humidity indicators. Very interestingly, ambient operation and shelf‐life stability are observed for devices based on nonenvironmentally stable semiconducting polymers, denoting an effective encapsulation capability of honey. Such effect would favor the development and application of future edible electronic circuits and systems. To this end, we also showed the compatibility of HGOFETs with release on food stuff from temporary paper‐tattoo substrates. While the edibility of the adopted semiconducting polymers has not been assessed yet, with only biocompatibility and/or cytotoxicity data available, the latter demonstration offers a proof‐of‐concept platform for future low‐power edible circuits.

Ongoing studies are focused on the first assessment of edibility of the employed active materials through in vitro digestion tests, and on the investigation of alternative semiconductors, such as natural dyes, for which edibility is already demonstrated.

We believe that such “sweet” and functional honey‐based devices hold a considerable promise for future development of edible electronics, and its integration to the healthcare sector.

## 4 Experimental Section

### Materials

Semiconducting polymers regioregular P3HT (regioregularity >99%; *M*_W_ = 17.5 kDa) and P(NDI2OD‐2T) (Activink N2200, *M*_n_ = 26.6 kDa and polydispersity (PDI) of 3.2) were purchased from Sigma‐Aldrich and Flexterra Inc., respectively, and used as received without any further purification. Honey (Millefiori della Patagonia, LOT 19248‐2, Eridania) was purchased from the supermarket, and used as is, being stored in the ambient environment. Commercial tattoo‐paper (Tattoo 2.1) was acquired from The Magic Touch Ltd. and used without any cleaning or surface treatment, apart from dust removal by nitrogen flushing.

### Sample Preparation

P3HT was dissolved in chlorobenzene (2.6 mg mL^−1^) and P(NDI2OD‐2T) was dissolved in toluene (5 mg mL^−1^) by stirring the solutions at ≈80 °C and room temperature, respectively. The inks were regularly stirred at the corresponding temperatures for 20 min.

For HGOFETs, a bottom‐contact architecture was adopted. Thoroughly cleaned corning 1737F glass (sonication in acetone and isopropanol, followed by 10 min of oxygen plasma) was used as the substrate for the first set of experiments. Interdigitated Au source and drain contacts of *L* =10 µm and *W* = 20 000 µm were defined by a lift‐off photolithographic process. A Cr adhesion layer (thickness: ≈1 nm) and a Au layer (32 nm) were thermally evaporated. The substrates were rinsed with isopropanol before deposition of the semiconductor. The formulations of P3HT and P(NDI2OD‐2T) were deposited by spin‐coating at 1000 rpm for 30 s in air, and finally annealed at 90 and 120 °C, correspondingly, for 20 min in an inert environment to remove residual solvents. The honey was then drop‐cast onto the channel area, and a tungsten probe was immersed in honey to serve as a gate electrode. The HGOFETs‐based inverting logic gate and ring oscillator were fabricated with connections realized with silver paste or wires.

Another set of HGOFETs was assembled directly onto commercial tattoo‐paper substrate. Au source and drain contacts were deposited through a shadow mask by vacuum thermal evaporation to achieve *L* =100 µm and *W* = 20 000 µm. The P3HT formulation was inkjet‐printed directly onto the contact and channel area with the Fujifilm Dimatix, DMP2831, and annealed at ≈120 °C for 20 min in inert environment. A honey drop was then cast, with tungsten probe served as a gate electrode. Transfer of the devices on food surfaces was performed by soaking the tattoo‐paper substrate in Milli Q water to achieve the dissolution of the sacrificial starch/dextrin layer, and subsequent release of EC onto the target item by sliding it off the paper carrier. A honey drop was then cast onto the transferred device.

### Device Characterization

Measurements of the HGOFETs and HGOFET‐based circuits’ characteristic curves were performed both in air and in inert atmosphere (nitrogen‐filled glovebox, O_2_, and H_2_O levels below 1 ppm) by means of an Agilent B1500A Semiconductor Parameter Analyzer. All the devices were measured in “yellow” room under the light filtered below 500 nm. The devices were periodically exposed to the microscope white light in order to probe the sample. Mobility–capacitance product values and threshold voltages were obtained by a linear fitting of *I*_ds_ or its square‐root for linear or saturation regimes, respectively.

Capacitance per unit area of the honey/semiconductor interface was extracted from Au/honey/semiconductor/Au configurations by means of EIS at 0.1 Hz with a potentiostat (Metrohm Autolab PGstat 302N, Nova 1.8 software) working in a two‐electrode configuration.

In order to avoid the contribution of Au counter‐electrode in the measured complex impedance, its surface area (≈7 cm^2^) was selected to significantly exceed the contact area of the working electrode with semiconductor/Au interface, ≈5 mm^2^. The impedance spectra were recorded in the frequency range from 0.001 Hz to 100 kHz by applying a continuous bias of ±0.4 V.

## Conflict of Interest

The authors declare no conflict of interest.

## Supporting information

Supporting information

## Data Availability

Research data are not shared.
