# Supplementary material for: Sweet Electronics: Honey‐Gated Complementary Organic Transistors and Circuits Operating in Air
Source: Adv Mater. 2021 Aug 21;33(40):2103183. doi: 10.1002/adma.202103183 (PMC11468742; doi:10.1002/adma.202103183)
Supplement: Supplementary file 1 — Supporting information [file ADMA-33-2103183-s001.pdf]

© 2021 Wiley-VCH GmbH

ADVANCED MATERIALS

Supporting Information

for *Adv. Mater.,* DOI: 10.1002/adma.202103183

Sweet Electronics: Honey-Gated Complementary Organic Transistors and Circuits Operating in Air

*Alina S. Sharova and Mario Caironi**

Supporting Information

Sweet electronics: honey-gated complementary organic transistors and circuits operating in air

*Alina S. Sharova, Mario Caironi**

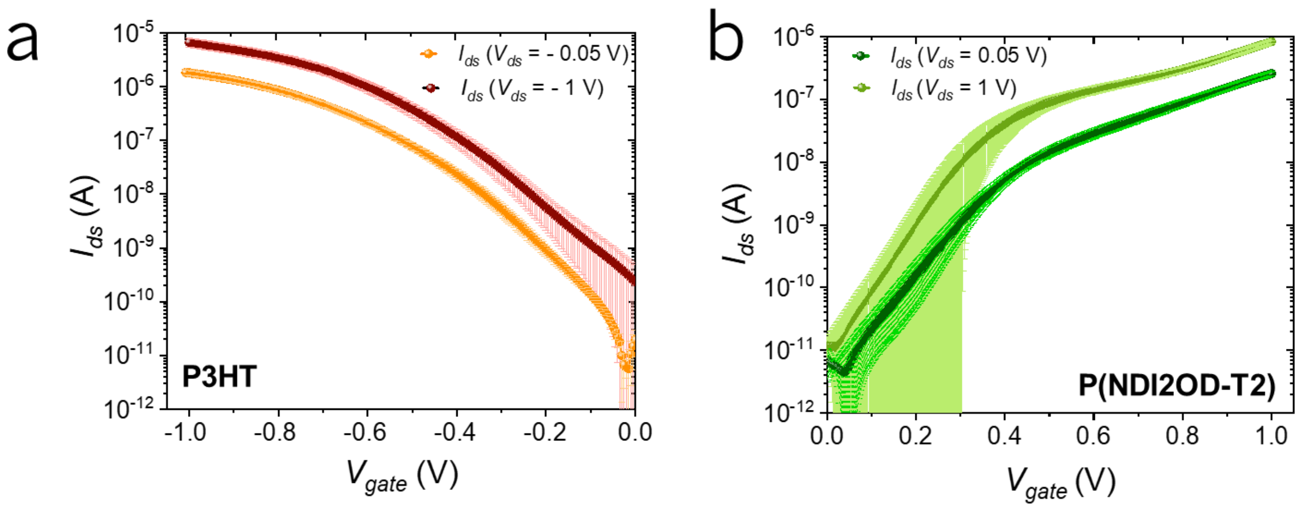

Figure S1. Reproducibility of the HGOFETs. Average transfer characteristic curves among 6 samples for **(a)** p-type HGOFETs in linear (V_{ds} = - 0.05 V) and saturation (V_{ds} = - 1 V) regimes and **(b)** n-type HGOFETs in linear (V_{ds} = 0.05 V) and saturation (V_{ds} =1 V) regimes; HGOFETs geometrical parameters: L =10 µm; W = 20,000 µm. Shaded area represents standard deviation.

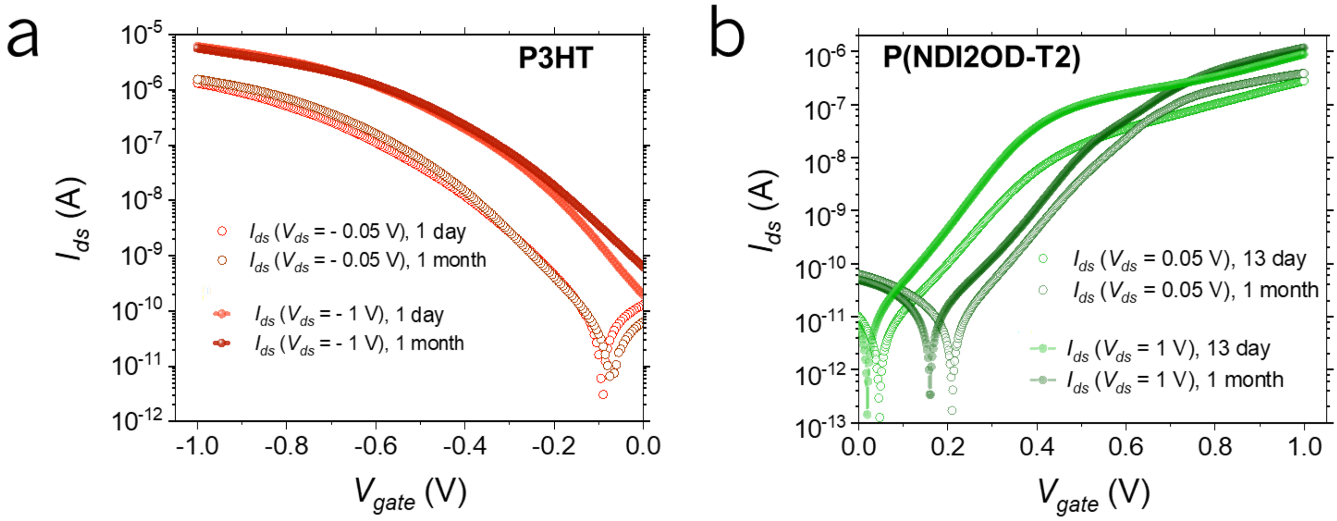

Figure S2. Shelf-life stability of the **(a)** p-type and **(b)** n-type HGOFETs. Comparison of electronic performances (transfer characteristics in linear and saturation regimes) after 1-month control period of storing the devices in air. The relative humidity varied from 48.8 to

50.4 %, what explains the threshold voltage shift of P(NDI2OD-T2)-based devices to higher values.

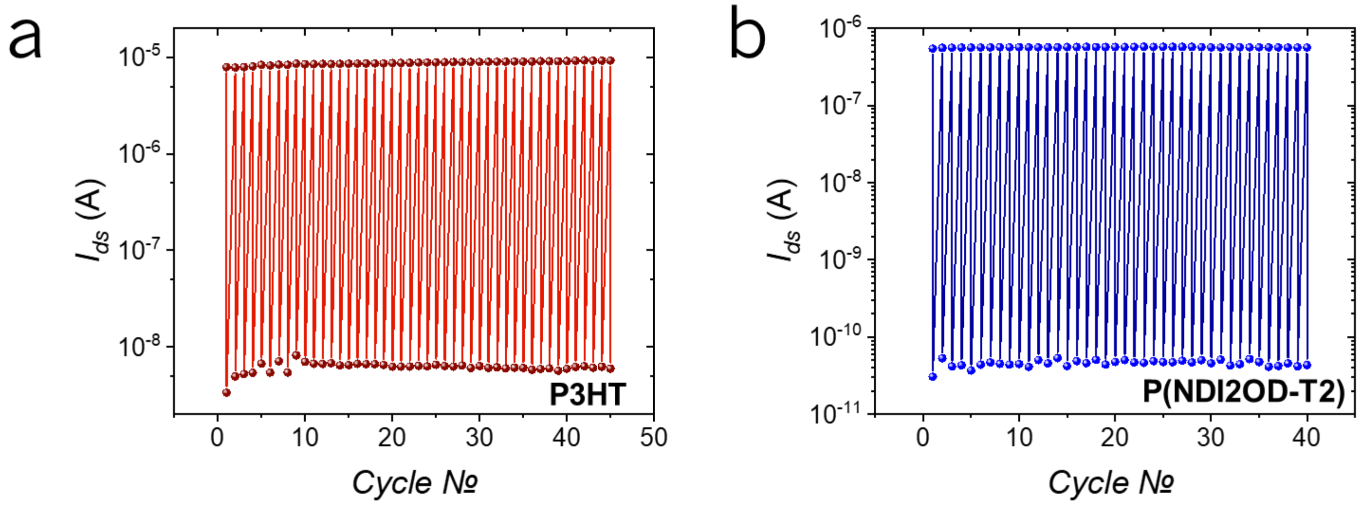

Figure S3. Operational stability of HGOFETs under continuous cycling voltage test in air (cycle duration 40 s, total test duration 30 min). The test was carried out by switching **(a)** the p-type device on (V_{gate} = -1 V, V_{ds} = - 0.2 V;) and off (V_{gate} = 0 V, V_{ds} = 0.2 V) and **(b)** n-type HGOFETs on (V_{gate} = 1 V, V_{ds} = 0.2 V) and off (V_{gate} = 0 V, V_{ds} = - 0.2 V). No evidence of devices degradation was observed.

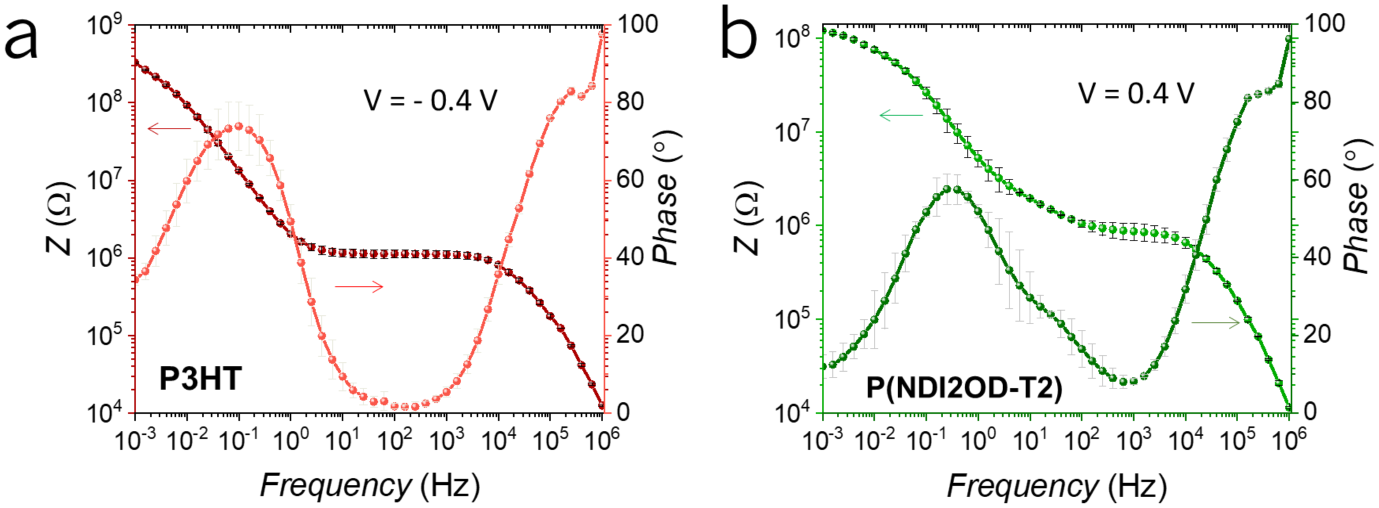

Figure S4. EIS characterization of Au/honey/semiconductor/Au structures based on **(a)** P3HT- and **(b)** P(NDI2OD-T2) (Au counter-electrode voltage, ±0.4 V, semiconductor/Au interface area ~5 mm^2; Au counter-electrode area ~7 cm^2). Average impedance and phase angle plots among 3 samples. "Hold-time" of 120 s is set in order to reach a stability. Sampling: 5 points for each decade.

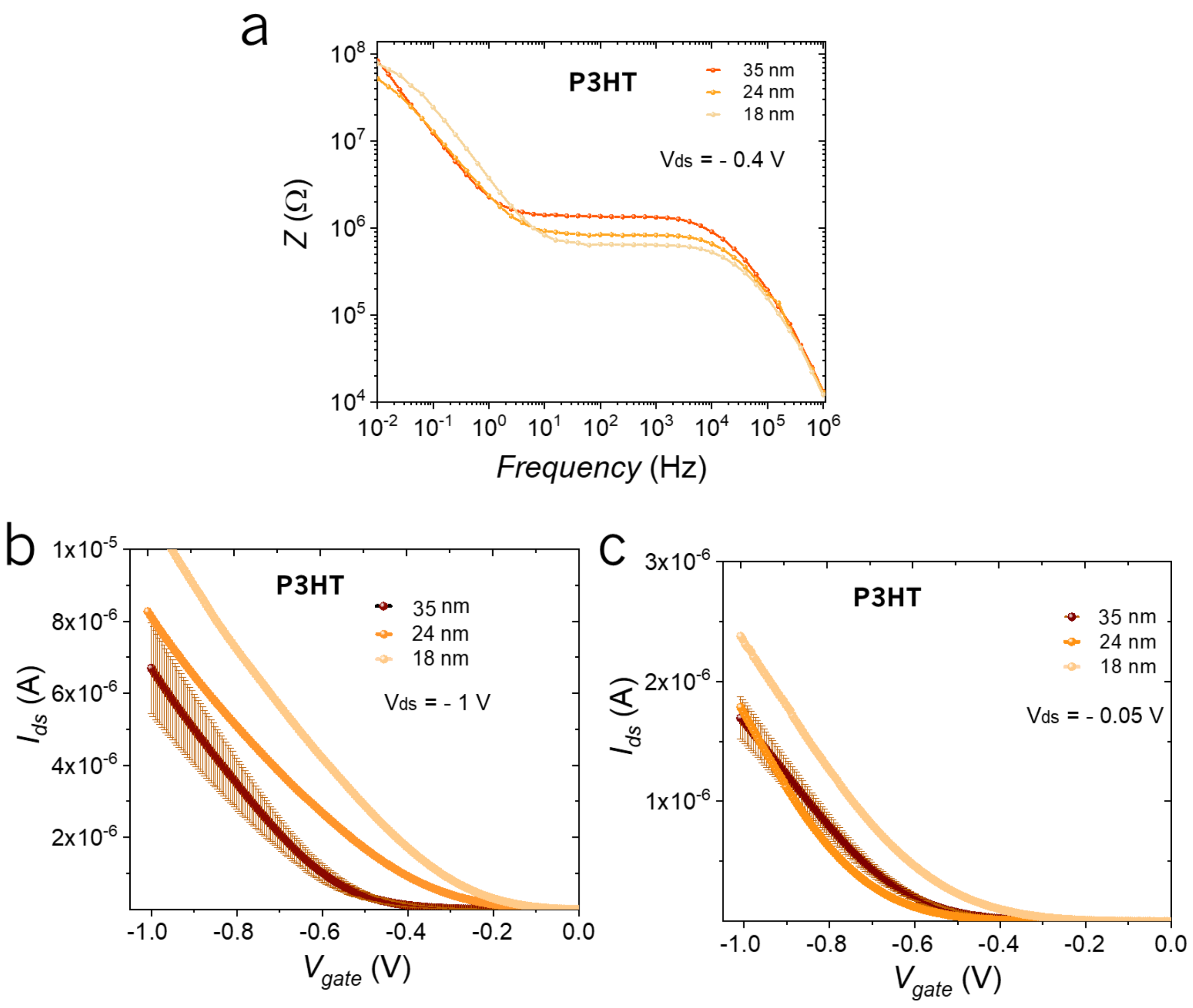

Figure S5. The semiconductor thickness dependence of the electrical performance of p-type devices. **(a)** EIS characterization of Au/honey/semiconductor/Au structures based on P3HT (Au counter-electrode voltage, -0.4 V, semiconductor/Au interface area ~5 mm^2; Au counter-electrode area ~7 cm^2). **(b)** Transfer characteristic curves in saturation (V_{ds} = - 1 V) and **(c)** linear (V_{ds} = - 0.05 V) regimes.

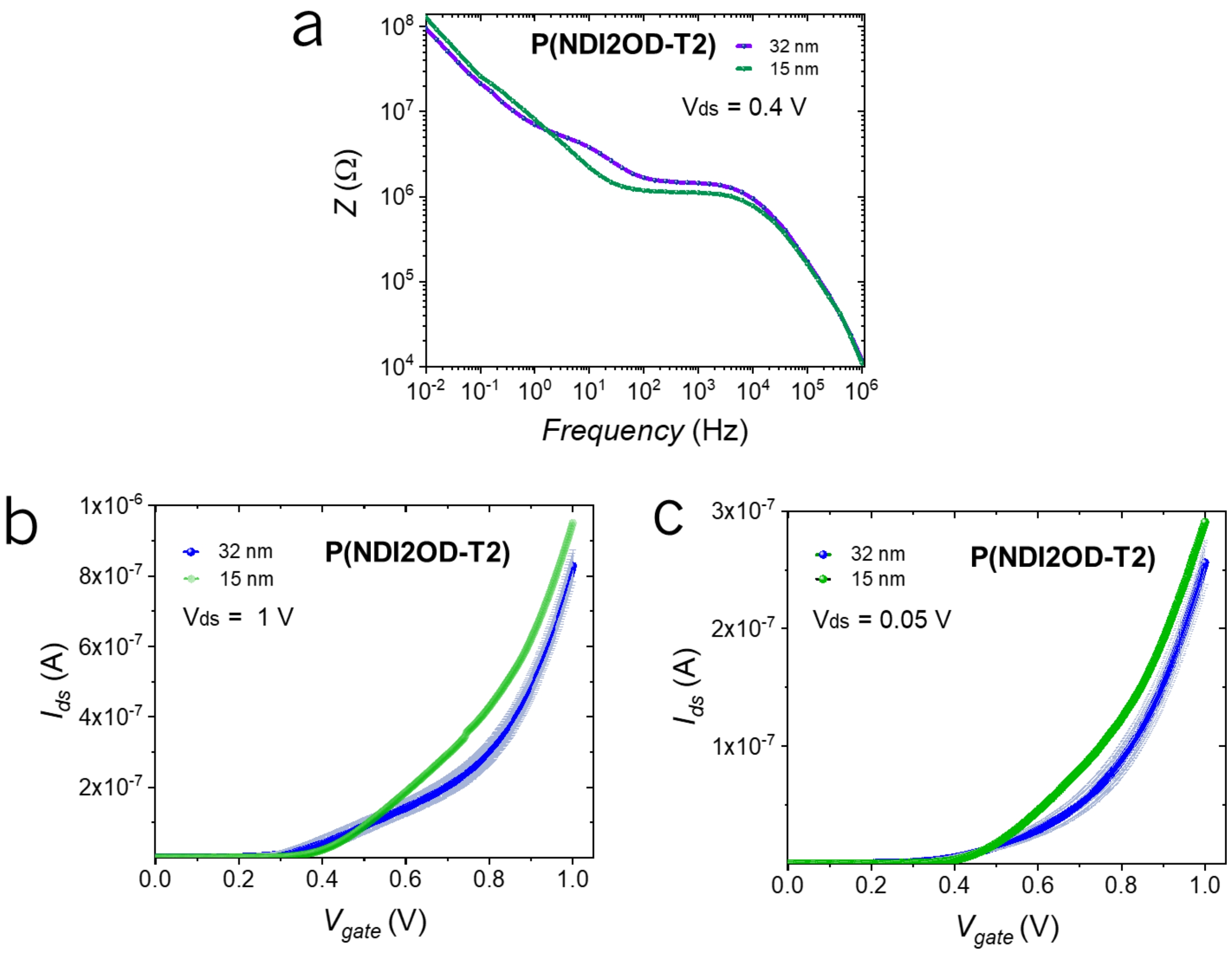

Figure S6. The semiconductor thickness dependence of the electrical performance of n-type devices. **(a)** EIS characterization of Au/honey/semiconductor/Au structures based on P(NDI2OD-T2) (Au counter-electrode voltage, 0.4 V, semiconductor/Au interface area ~5 mm^2; Au counter-electrode area ~7 cm^2). **(b)** Transfer characteristic curves in saturation (V_{ds} = 1 V) and **(c)** linear (V_{ds} = 0.05 V) regimes.

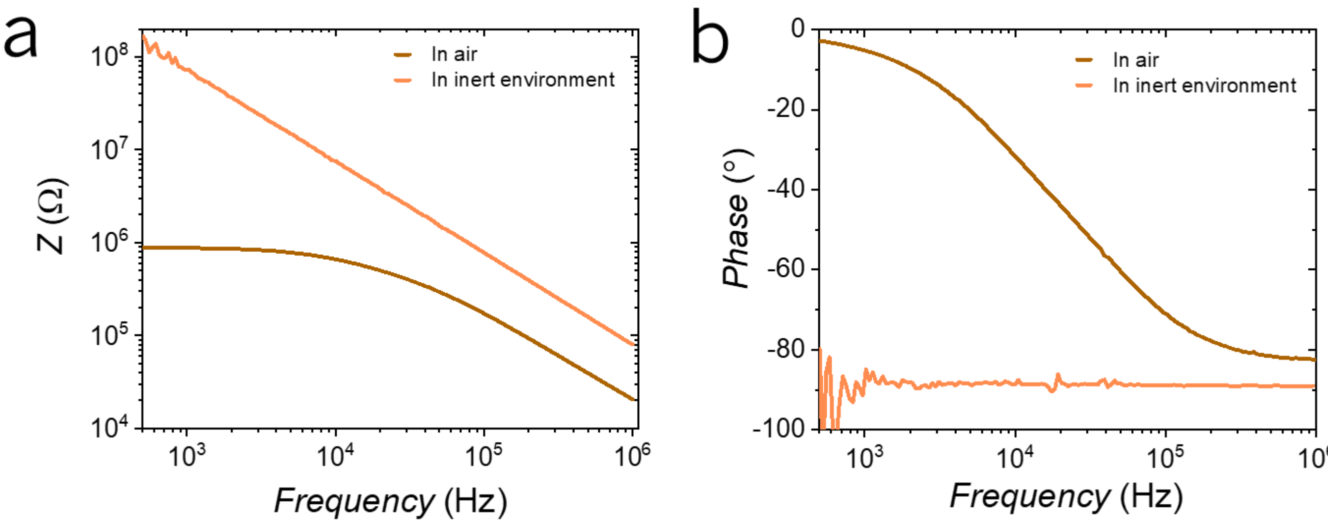

Figure S7. EIS characterization of Au/honey/semiconductor/Au structures based on P3HT in air and in inert environment (Au counter-electrode voltage, -0.4 V, semiconductor/Au interface area ~5 mm^2; Au counter-electrode area ~7 cm^2)

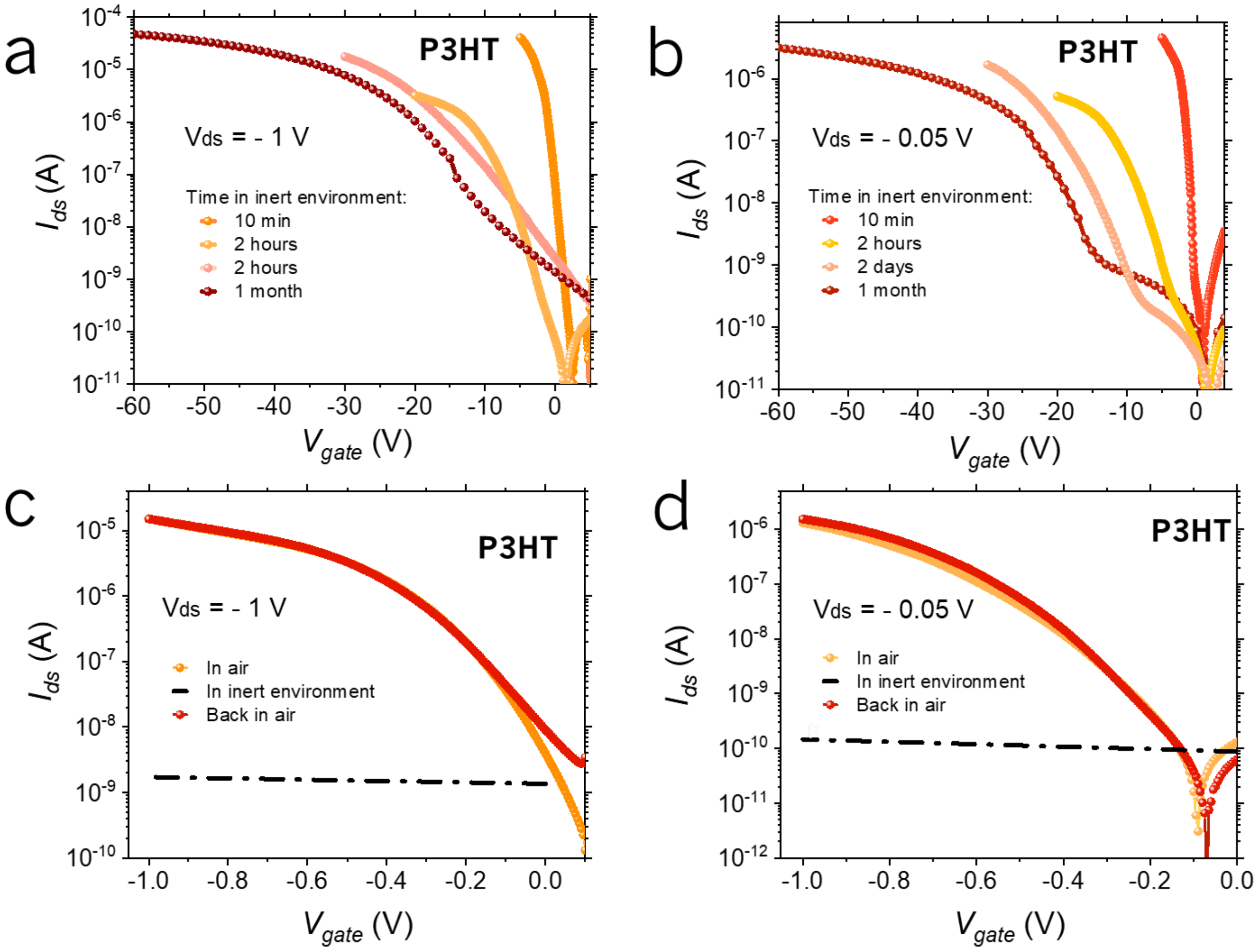

Figure S8. The electrical response of p-type HGOFETs to different humidity conditions (relative humidity in air ≈ 50 %; in inert nitrogen environment ≈ 0 %). The humidity-dependent transfer characteristics of p-type HGOFETs operating in both **(a, c)** saturation (V_{ds} = - 1 V) and **(b, d)** linear (V_{ds} = - 0.05 V) regimes.

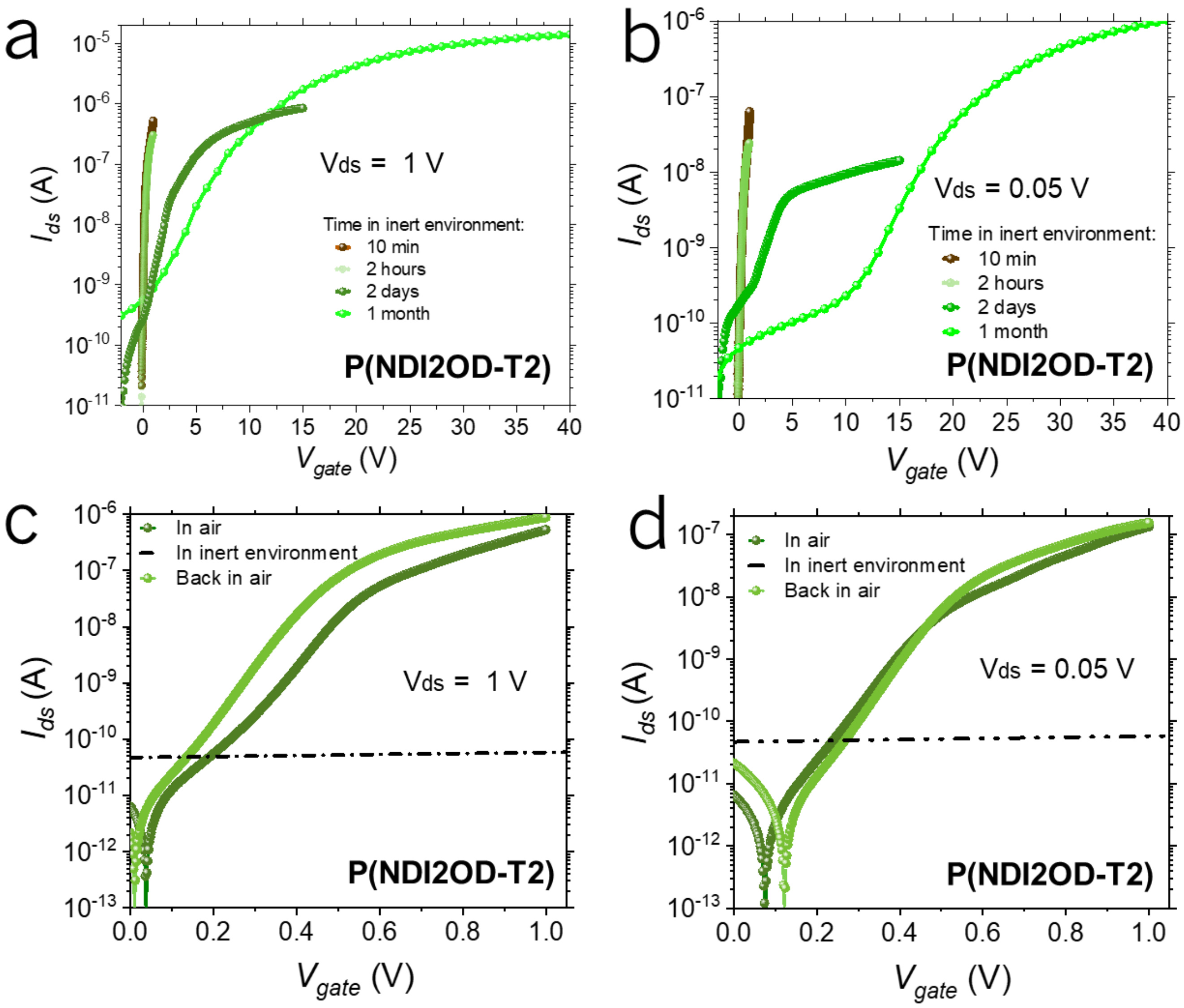

Figure S9. The electrical response of n-type HGOFETs to different humidity conditions (relative humidity in air ≈ 50 %; in inert nitrogen environment ≈ 0 %). The humidity-dependent transfer characteristics of n-type HGOFETs operating in both **(a, c)** saturation (V_{ds} = 1 V) and **(b, d)** linear (V_{ds} = 0.05 V) regimes.

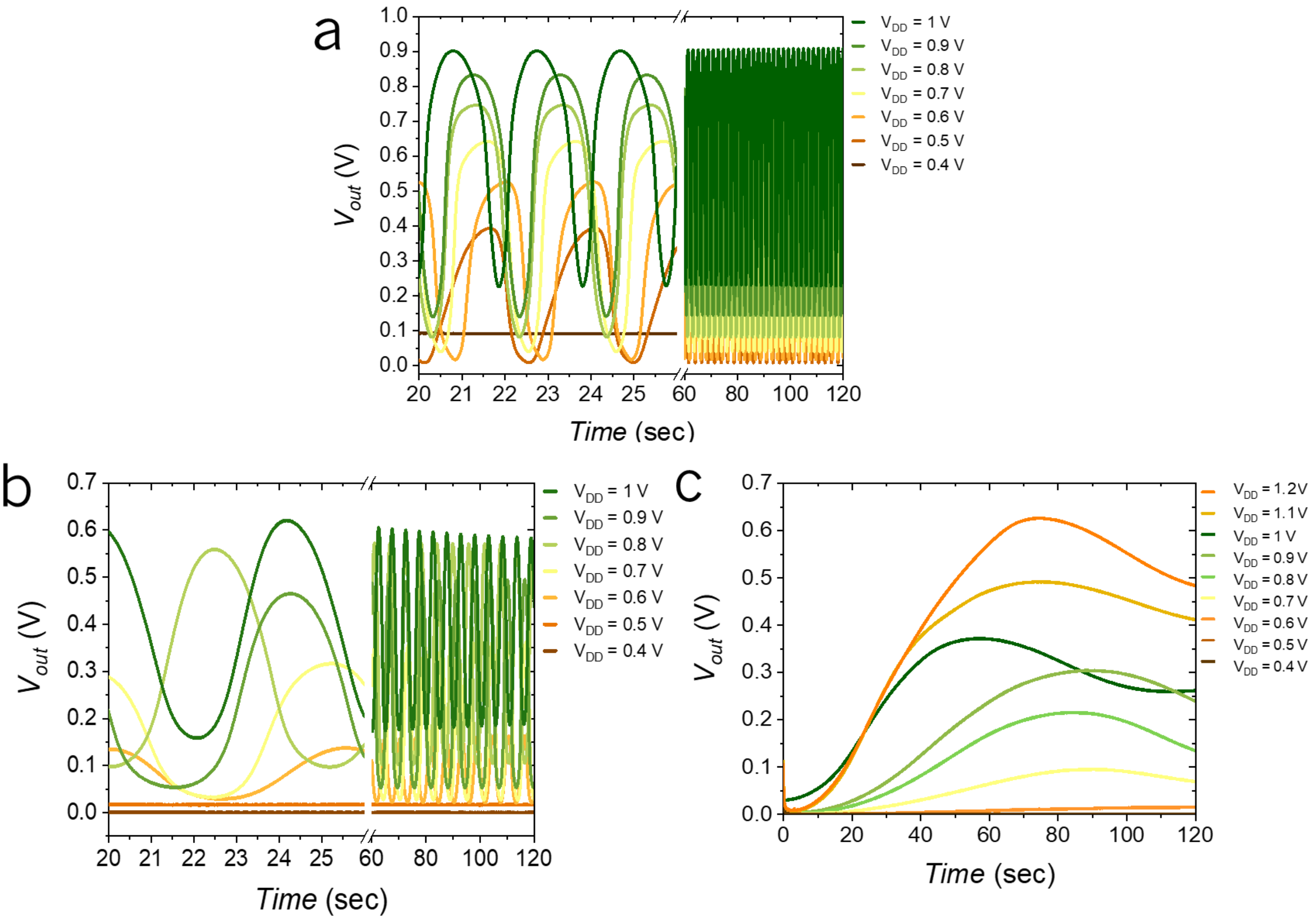

Figure S10. The output waveforms of the HGOFETs-based oscillator at different supply voltages V_{DD} after **(a)** 10 min; **(b)** 2 days; **(c)** 4 days after exposing the device to inert environment.

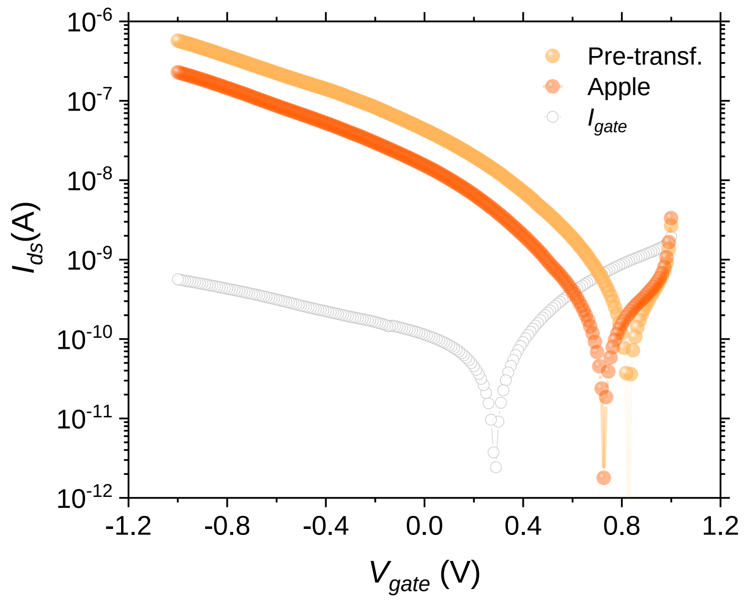

Figure S11. HGOFETs on flexible edible tattoo-paper substrate. Transfer characteristic of p-type HGOFET in saturation ($V_{ds} = -0.7$ V) regime before and after the transfer onto the apple. (Honey was drop-cast onto the channel area after the transfer procedure)
